# 4-[(*E*)-(4-Eth­oxy­phen­yl)imino­meth­yl]phenol

**DOI:** 10.1107/S1600536812034253

**Published:** 2012-08-04

**Authors:** Aliakbar Dehno Khalaji, Karla Fejfarová, Michal Dušek

**Affiliations:** a Department of Chemistry, Faculty of Science, Golestan University, Gorgan, Iran; b Institute of Physics ASCR, v.v.i., Na Slovance 2, 182 21 Praha 8, Czech Republic

## Abstract

In the title compound, C_15_H_15_NO_2_, the dihedral angle between the benzene rings is 52.04 (5)° and the mol­ecule has an *E* conformation about the central C=N bond. In the crystal, mol­ecules are connected by O—H⋯N hydrogen bonds, forming zigzag chains along the *b* axis. The crystal packing also features weak C—H⋯O inter­actions.

## Related literature

For Schiff base derivatives and related structures, see: Fejfarová *et al.* (2010 ▶); Özek *et al.* (2010 ▶); Akkurt *et al.* (2008 ▶); Khalaji *et al.* (2008 ▶, 2009 ▶) For applications and properties of Schiff bases, see: Dalapati *et al.* (2011 ▶); Keypour *et al.* (2010 ▶); Khalil *et al.* (2009 ▶); Khanmohammadi *et al.* (2009 ▶); Sun *et al.* (2012 ▶); Da Silva *et al.* (2011 ▶).
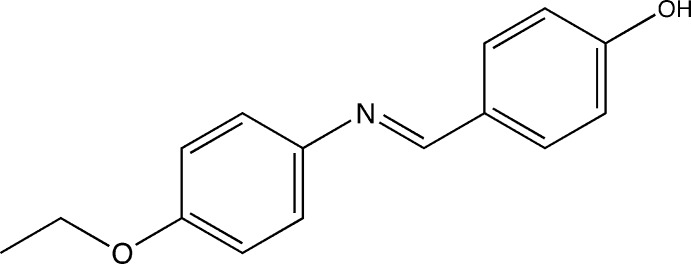


## Experimental

### 

#### Crystal data

C_15_H_15_NO_2_
*M* *_r_* = 241.3
Orthorhombic, 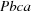

*a* = 10.9155 (2) Å
*b* = 9.4056 (2) Å
*c* = 25.0422 (5) Å
*V* = 2571.00 (9) Å^3^
*Z* = 8
Cu *K*α radiation
μ = 0.67 mm^−1^
*T* = 120 K
0.54 × 0.20 × 0.03 mm

#### Data collection

Agilent Xcalibur diffractometer with an Atlas (Gemini ultra Cu) detector
Absorption correction: multi-scan (*CrysAlis PRO*; Agilent, 2011 ▶) *T*_min_ = 0.795, *T*_max_ = 1
22644 measured reflections
1978 independent reflections
1780 reflections with *I* > 3σ(*I*)
*R*_int_ = 0.028
θ_max_ = 61.1°

#### Refinement

*R*[*F* ^2^ > 3σ(*F* ^2^)] = 0.031
*wR*(*F* ^2^) = 0.103
*S* = 1.71
1978 reflections
166 parameters
H atoms treated by a mixture of independent and constrained refinement
Δρ_max_ = 0.11 e Å^−3^
Δρ_min_ = −0.11 e Å^−3^

### 

Data collection: *CrysAlis PRO* (Agilent, 2011 ▶); cell refinement: *CrysAlis PRO*; data reduction: *CrysAlis PRO*; program(s) used to solve structure: *SIR2002* (Burla *et al.*, 2003 ▶); program(s) used to refine structure: *JANA2006* (Petříček *et al.*, 2006 ▶); molecular graphics: *DIAMOND* (Brandenburg & Putz, 2005 ▶); software used to prepare material for publication: *JANA2006*.

## Supplementary Material

Crystal structure: contains datablock(s) global, I. DOI: 10.1107/S1600536812034253/bt5991sup1.cif

Structure factors: contains datablock(s) I. DOI: 10.1107/S1600536812034253/bt5991Isup2.hkl

Supplementary material file. DOI: 10.1107/S1600536812034253/bt5991Isup3.cml

Additional supplementary materials:  crystallographic information; 3D view; checkCIF report

## Figures and Tables

**Table 1:** Hydrogen-bond geometry (Å, °)

*D*—H⋯*A*	*D*—H	H⋯*A*	*D*⋯*A*	*D*—H⋯*A*
C7—H7*b*⋯O2^i^	0.96	2.50	3.3393 (15)	147
O2—H2*o*⋯N1^ii^	0.894 (17)	1.825 (17)	2.7098 (12)	170.0 (14)

Symmetry codes: (i) 
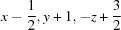
; (ii) 
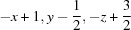
.

**Fig. 1.**
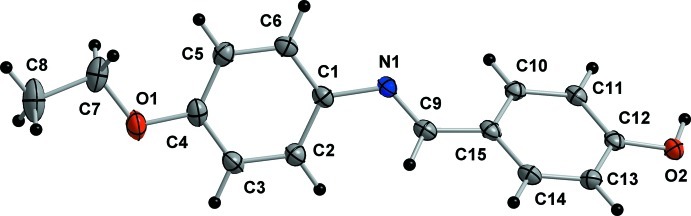
Molecular structure of the title compound. Displacement ellipsoids are shown at the 50% probability level.

**Fig. 2.**
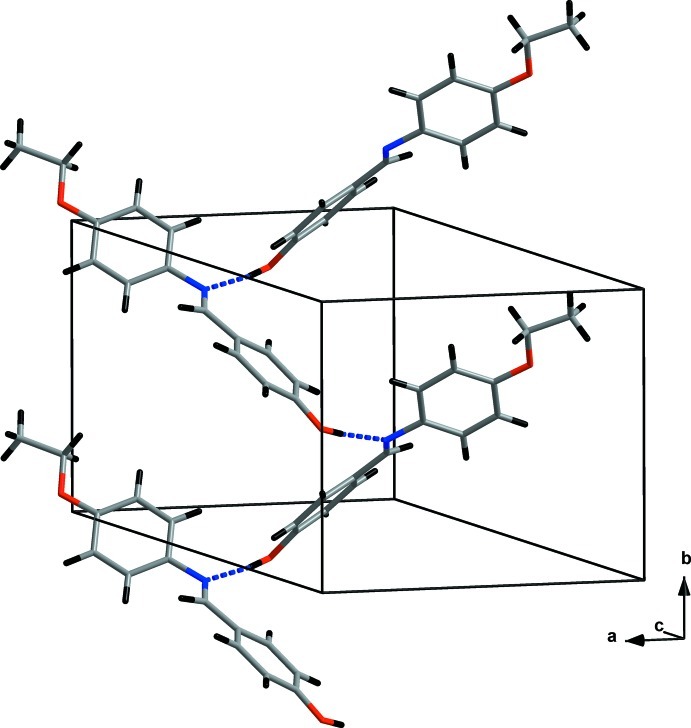
Hydrogen-bonded chain propagating along the b axis. Hydrogen bonds are drawn as dashed lines.

## supplementary crystallographic information

### Comment

Schiff base compounds exhibit a broad range of biological activities, including antifungal, and antibacterial (Da Silva *et al.*, 2011). They are used as anion sensors (Dalapati *et al.*, 2011; Khalil *et al.*, 2009), non-linear optics compounds (Sun *et al.*, 2012), and as versatile ligands in coordination chemistry (Khanmohammadi *et al.*, 2009; Keypour *et al.*, 2010). The present work is part of a structural study of Schiff bases (Khalaji *et al.*, 2008, 2009; Fejfarová *et al.*, 2010) and we report here the structure of (*E*)-(4-hydroxybenzylidene)-4-ethoxyaniline, (1).

The molecule of (1) (Fig. 1) has an *E* conformation about the central C=N bond and the C=N and C—N bond lengths of 1.2853 (45) and 1.4250 (14) Å agree well with the corresponding distances in other Schiff bases (Akkurt *et al.*, 2008; Özek *et al.*, 2010; Khalaji *et al.*, 2008, 2009; Fejfarová *et al.*, 2010). The dihedral angle between the two benzene rings is 52.04 (5)°. The ethoxy group is almost coplanar with the adjacent ring [dihedral angle 3.51 (12)°]. The molecules are connected by intermolecular O—H···N hydrogen bonds, forming zigzag chains along the *b* axis (Fig. 2). The crystal structure is further stabilized by intermolecular C—H···O hydrogen bonds.

### Experimental

To a stirring solution of the 4-hydroxybenzaldehyde (0.2 mmol, in 5 ml of methanol) was added 4-ethoxyaniline (0.2 mmol) in 10 ml of methanol and the mixture was stirred for 1 h in air at 323 K and was then left at room temperature for several days without disturbance yielding suitable crystals of 1 that subsequently were filtered off and washed with Et2O. Yield: 82%.

### Refinement

The hydroxyl hydrogen atom was found in difference Fourier maps and its coordinates were refined. All other hydrogen atoms were calculated geometrically and refined as riding on their parent atoms. The methyl H atoms were allowed to rotate freely about the adjacent C–C bond. The displacement coefficients of hydrogen atoms *U*_iso_(H) were set to 1.5*U*_eq_(C, O) for the methyl- and hydroxyl- groups and to to 1.2*U*_eq_(C) for the CH– and CH2- groups.

### Crystal data

**Table:**

C_15_H_15_NO_2_	*F*(000) = 1024
*M**_r_* = 241.3	*D*_x_ = 1.246 Mg m^−^^3^
Orthorhombic, *P**b**c**a*	Cu *K*α radiation, λ = 1.5418 Å
Hall symbol: -P 2ac 2ab	Cell parameters from 14821 reflections
*a* = 10.9155 (2) Å	θ = 3.5–61.1°
*b* = 9.4056 (2) Å	µ = 0.67 mm^−^^1^
*c* = 25.0422 (5) Å	*T* = 120 K
*V* = 2571.00 (9) Å^3^	Plate, light yellow
*Z* = 8	0.54 × 0.20 × 0.03 mm

### Data collection

**Table:**

Agilent Xcalibur diffractometer with an Atlas (Gemini ultra Cu) detector	1978 independent reflections
Radiation source: Enhance Ultra (Cu) X-ray Source	1780 reflections with *I* > 3σ(*I*)
Mirror monochromator	*R*_int_ = 0.028
Detector resolution: 10.3784 pixels mm^-1^	θ_max_ = 61.1°, θ_min_ = 3.5°
Rotation method data acquisition using ω scans	*h* = −12→12
Absorption correction: multi-scan (*CrysAlis PRO*; Agilent, 2011)	*k* = −10→10
*T*_min_ = 0.795, *T*_max_ = 1	*l* = −28→27
22644 measured reflections	

### Refinement

**Table:**

Refinement on *F*^2^	57 constraints
*R*[*F*^2^ > 2σ(*F*^2^)] = 0.031	H atoms treated by a mixture of independent and constrained refinement
*wR*(*F*^2^) = 0.103	Weighting scheme based on measured s.u.'s *w* = 1/(σ^2^(*I*) + 0.0025000002*I*^2^)
*S* = 1.71	(Δ/σ)_max_ = 0.006
1978 reflections	Δρ_max_ = 0.11 e Å^−^^3^
166 parameters	Δρ_min_ = −0.11 e Å^−^^3^
0 restraints	

### Special details

**Table:**

Experimental. Absorption correction: CrysAlis PRO (Agilent, 2011). Empirical absorption correction using spherical harmonics, implemented in SCALE3 ABSPACK scaling algorithm.
Refinement. The refinement was carried out against all reflections. The conventional *R*-factor is always based on *F*. The goodness of fit as well as the weighted *R*-factor are based on *F* and *F*^2^ for refinement carried out on *F* and *F*^2^, respectively. The threshold expression is used only for calculating *R*-factors *etc*. and it is not relevant to the choice of reflections for refinement.The program used for refinement, Jana2006, uses the weighting scheme based on the experimental expectations, see _refine_ls_weighting_details, that does not force *S* to be one. Therefore the values of *S* are usually larger than the ones from the *SHELX* program.

### Fractional atomic coordinates and isotropic or equivalent isotropic displacement parameters (Å^2^)

**Table:**

	*x*	*y*	*z*	*U*_iso_*/*U*_eq_	
O1	−0.08099 (8)	0.58106 (10)	0.55939 (4)	0.0344 (3)	
O2	0.50129 (8)	−0.13939 (9)	0.85269 (3)	0.0251 (3)	
N1	0.26997 (8)	0.30259 (10)	0.68326 (4)	0.0196 (3)	
C1	0.18053 (10)	0.37189 (12)	0.65100 (4)	0.0200 (3)	
C2	0.06035 (11)	0.32217 (13)	0.64660 (4)	0.0234 (4)	
C3	−0.02398 (11)	0.39425 (13)	0.61577 (5)	0.0260 (4)	
C4	0.00974 (12)	0.51683 (14)	0.58837 (4)	0.0259 (4)	
C5	0.12999 (11)	0.56466 (13)	0.59109 (5)	0.0251 (4)	
C6	0.21487 (11)	0.49095 (12)	0.62174 (5)	0.0228 (4)	
C7	−0.04877 (15)	0.70560 (14)	0.52934 (5)	0.0357 (4)	
C8	−0.16378 (16)	0.75963 (18)	0.50327 (7)	0.0509 (5)	
C9	0.23493 (11)	0.24457 (11)	0.72715 (5)	0.0208 (4)	
C10	0.42099 (10)	0.09397 (12)	0.74119 (5)	0.0198 (4)	
C11	0.48630 (10)	−0.00196 (12)	0.77159 (4)	0.0199 (4)	
C12	0.44379 (10)	−0.04176 (12)	0.82220 (4)	0.0205 (4)	
C13	0.33635 (10)	0.01912 (12)	0.84201 (5)	0.0230 (4)	
C14	0.27084 (11)	0.11386 (12)	0.81106 (4)	0.0228 (4)	
C15	0.31179 (10)	0.15335 (12)	0.76005 (4)	0.0202 (4)	
H2	0.036296	0.23732	0.665154	0.028*	
H3	−0.106486	0.35958	0.613196	0.0313*	
H5	0.154298	0.648332	0.571861	0.0301*	
H6	0.298437	0.52267	0.622731	0.0273*	
H7a	0.009871	0.680691	0.502344	0.0429*	
H7b	−0.017107	0.776855	0.553066	0.0429*	
H8a	−0.144624	0.840538	0.481436	0.0764*	
H8b	−0.198521	0.686045	0.48144	0.0764*	
H8c	−0.221636	0.786824	0.530273	0.0764*	
H9	0.152732	0.262562	0.738996	0.0249*	
H10	0.450945	0.120386	0.706538	0.0237*	
H11	0.561133	−0.041581	0.758022	0.0239*	
H13	0.308026	−0.0049	0.877165	0.0276*	
H14	0.196003	0.153391	0.82466	0.0273*	
H2o	0.5725 (16)	−0.1625 (16)	0.8374 (6)	0.0377*	

### Atomic displacement parameters (Å^2^)

**Table:**

	*U*^11^	*U*^22^	*U*^33^	*U*^12^	*U*^13^	*U*^23^
O1	0.0303 (5)	0.0399 (6)	0.0330 (5)	0.0097 (4)	−0.0083 (4)	0.0063 (4)
O2	0.0188 (5)	0.0286 (5)	0.0279 (5)	0.0040 (3)	0.0011 (3)	0.0074 (3)
N1	0.0168 (5)	0.0182 (5)	0.0238 (5)	−0.0005 (4)	−0.0022 (4)	−0.0012 (4)
C1	0.0181 (6)	0.0208 (6)	0.0210 (6)	0.0025 (4)	0.0003 (4)	−0.0033 (5)
C2	0.0218 (6)	0.0235 (7)	0.0248 (6)	−0.0005 (5)	−0.0009 (5)	−0.0002 (5)
C3	0.0180 (6)	0.0325 (7)	0.0277 (7)	0.0002 (5)	−0.0020 (5)	−0.0016 (5)
C4	0.0263 (7)	0.0303 (7)	0.0212 (6)	0.0081 (5)	−0.0028 (5)	−0.0019 (5)
C5	0.0281 (7)	0.0243 (6)	0.0229 (6)	0.0017 (5)	0.0022 (5)	0.0005 (5)
C6	0.0204 (6)	0.0238 (6)	0.0241 (6)	−0.0003 (5)	0.0014 (5)	−0.0020 (5)
C7	0.0496 (9)	0.0313 (7)	0.0262 (7)	0.0121 (6)	−0.0067 (6)	0.0008 (5)
C8	0.0644 (11)	0.0465 (9)	0.0418 (9)	0.0231 (8)	−0.0190 (8)	−0.0003 (7)
C9	0.0166 (6)	0.0199 (6)	0.0259 (7)	−0.0002 (4)	−0.0001 (5)	−0.0030 (5)
C10	0.0176 (6)	0.0207 (6)	0.0209 (6)	−0.0030 (4)	−0.0007 (4)	−0.0011 (4)
C11	0.0154 (6)	0.0205 (6)	0.0239 (6)	−0.0006 (4)	−0.0010 (4)	−0.0023 (5)
C12	0.0169 (6)	0.0196 (6)	0.0249 (6)	−0.0035 (4)	−0.0041 (4)	−0.0005 (5)
C13	0.0190 (6)	0.0271 (7)	0.0228 (6)	−0.0026 (5)	0.0014 (5)	0.0015 (5)
C14	0.0167 (6)	0.0248 (6)	0.0267 (6)	0.0005 (5)	0.0007 (5)	−0.0014 (5)
C15	0.0171 (6)	0.0193 (6)	0.0243 (6)	−0.0030 (4)	−0.0026 (5)	−0.0025 (5)

### Geometric parameters (Å, º)

**Table:**

O1—C4	1.3684 (15)	C7—H7a	0.96
O1—C7	1.4360 (16)	C7—H7b	0.96
O2—C12	1.3492 (14)	C8—H8a	0.96
O2—H2o	0.894 (17)	C8—H8b	0.96
N1—C1	1.4250 (14)	C8—H8c	0.96
N1—C9	1.2853 (14)	C9—C15	1.4557 (16)
C1—C2	1.3971 (16)	C9—H9	0.96
C1—C6	1.3897 (16)	C10—C11	1.3791 (16)
C2—C3	1.3795 (17)	C10—C15	1.3985 (15)
C2—H2	0.96	C10—H10	0.96
C3—C4	1.3913 (17)	C11—C12	1.4006 (15)
C3—H3	0.96	C11—H11	0.96
C4—C5	1.3892 (17)	C12—C13	1.3961 (16)
C5—C6	1.3887 (16)	C13—C14	1.3807 (16)
C5—H5	0.96	C13—H13	0.96
C6—H6	0.96	C14—C15	1.4033 (16)
C7—C8	1.504 (2)	C14—H14	0.96
			
C4—O1—C7	117.43 (10)	C7—C8—H8a	109.47
C12—O2—H2o	109.1 (9)	C7—C8—H8b	109.47
C1—N1—C9	118.37 (9)	C7—C8—H8c	109.47
N1—C1—C2	122.33 (10)	H8a—C8—H8b	109.47
N1—C1—C6	118.86 (10)	H8a—C8—H8c	109.47
C2—C1—C6	118.78 (10)	H8b—C8—H8c	109.47
C1—C2—C3	120.40 (11)	N1—C9—C15	124.24 (10)
C1—C2—H2	119.8	N1—C9—H9	117.88
C3—C2—H2	119.8	C15—C9—H9	117.88
C2—C3—C4	120.45 (11)	C11—C10—C15	121.02 (10)
C2—C3—H3	119.77	C11—C10—H10	119.49
C4—C3—H3	119.77	C15—C10—H10	119.49
O1—C4—C3	115.84 (11)	C10—C11—C12	120.21 (10)
O1—C4—C5	124.54 (11)	C10—C11—H11	119.89
C3—C4—C5	119.61 (11)	C12—C11—H11	119.9
C4—C5—C6	119.73 (11)	O2—C12—C11	122.68 (10)
C4—C5—H5	120.14	O2—C12—C13	117.95 (10)
C6—C5—H5	120.14	C11—C12—C13	119.35 (10)
C1—C6—C5	120.91 (11)	C12—C13—C14	120.02 (11)
C1—C6—H6	119.54	C12—C13—H13	119.99
C5—C6—H6	119.54	C14—C13—H13	119.99
O1—C7—C8	107.38 (12)	C13—C14—C15	121.12 (10)
O1—C7—H7a	109.47	C13—C14—H14	119.44
O1—C7—H7b	109.47	C15—C14—H14	119.44
C8—C7—H7a	109.47	C9—C15—C10	122.37 (10)
C8—C7—H7b	109.47	C9—C15—C14	119.18 (10)
H7a—C7—H7b	111.48	C10—C15—C14	118.24 (10)
			
C4—O1—C7—C8	−177.51 (11)	N1—C9—C15—C10	−15.04 (18)
C9—N1—C1—C2	−35.10 (15)	N1—C9—C15—C14	170.16 (11)
C9—N1—C1—C6	146.67 (11)		

### Hydrogen-bond geometry (Å, º)

**Table:**

*D*—H···*A*	*D*—H	H···*A*	*D*···*A*	*D*—H···*A*
C7—H7*b*···O2^i^	0.96	2.50	3.3393 (15)	147
O2—H2*o*···N1^ii^	0.894 (17)	1.825 (17)	2.7098 (12)	170.0 (14)

Symmetry codes: (i) *x*−1/2, *y*+1, −*z*+3/2; (ii) −*x*+1, *y*−1/2, −*z*+3/2.
